# Metabolomic Profiling in Children with Sleep Disorders: An Exploratory Pilot Study

**DOI:** 10.3390/jcm15176595

**Published:** 2026-08-26

**Authors:** Roberta Pintus, M. Evangelisti, C. Piras, M. Arpinelli, F. Vassilios, A. Noto, G. Di Nardo, Maria Pia Villa

**Affiliations:** 1 Neonatal Intensive Care Unit, Department of Surgical Sciences, University Hospital of Cagliari, University of Cagliari, 09124 Cagliari, Italy; 2 Paediatrics Unit, Neurosciences, Mental Health, and Sense Organs (NESMOS) Department, St. Andrea University Hospital, Faculty of Medicine and Psychology, Sapienza University of Rome, 00185 Rome, Italy; 3 Clinical Metabolomics Unit, Department of Biomedical Sciences, University of Cagliari, Blocco A, 09124 Cagliari, Italy

**Keywords:** obstructive sleep apnea syndrome, sleep disorder, parasomnias, metabolomic analysis, children

## Abstract

**Objectives:** To analyze the metabolomic profile of children with sleep disorders to understand whether there are distinct and specific metabolic patterns related to the severity and type of sleep disorders (neurological or respiratory). **Materials and Methods:** This is an observational pilot study. Patients referred to our department for sleep disorders—both neurological and breath-related sleep disturbances—were enrolled. All patients received an anamnestic interview, clinical evaluation, polysomnographic sleep study and urine collection for the metabolomic analysis. **Results:** Obstructive sleep apnea (OSA) patients were divided into mild and moderate while the third group was represented by patients with parasomnias. The oxalic acid and ribose increased in moderate OSA and pavor compared to mild patients, while 3-hydroxypropionic decreased. Furthermore, the pyroglutamic acid, aminomalonica acid, urea, p-cresol, glycine, 4-hydroxyphenyl acetate, citric acid and ethanolamine increased in moderate OSA patients with respect to mild ones. Finally, hippuric acid and glutamic acid decreased in moderate patients compared to mild ones. The enrichment analysis revealed that urea cycle, tryptophan metabolism, glucose-alanine cycle, glutathione metabolism and beta-alanine metabolism were the most significantly discriminant pathways between OSA patients, while the pentose phosphate pathway, propanoate metabolism, Warburg effect, citric acid cycle and transfer of acetyl group into mitochondria were the most significantly discriminant pathways between mild OSA and pavor patients. **Conclusions:** our data reveal interesting correlations between metabolomics and sleep disorders that deserve further investigations in larger cohorts of patients and the integration of different approaches to enhance our understanding of the field and guide clinicians toward precise and personalized patient management.

## 1. Introduction

Pediatric sleep disorders encompass a wide spectrum of clinical manifestations, ranging from respiratory disorders, such as obstructive sleep apnea (OSA), to neurological disorders, such as parasomnias [1].

Sleep-disordered breathing is the most common sleep disorder in children [2].

Parasomnias are characterized by abnormal motor, verbal, or behavioral events occurring during sleep or sleep–wake transitions. According to the International Classification of Sleep Disorders-3 (ICSD-3), they are categorized into non-rapid eye movement (NREM)-related, rapid eye movement (REM)-related, and other parasomnias [3].

Pavor nocturnus (i.e., sleep terror) is an NREM-related parasomnia defined by sudden episodes of fear, screaming and crying during sleep in association with autonomic hyperactivity (tachycardia, tachypnea, sweating) in which patients appear inconsolable. Episodes are usually followed by a total or partial amnesia of the event [4]. The gold standard for sleep disorder diagnosis is polysomnography, which allows for the classification of the type of sleep disorder and the assessment of its severity.

OSA is characterized by upper airway obstructions that determine a reduction in oxygen supply, triggering intermittent hypoxia phenomena with subsequent reoxygenation, capable of generating oxidative stress through the production of Reactive Oxygen Species (ROS), responsible for damage to the entire organism due to systemic inflammation. For this reason, sleep-related breathing disorders are considered disorders with systemic impact and, if not adequately treated, are related to important cardiological, endocrinological and neurobehavioral comorbidities [5,6,7].

This is why early recognition and treatment is mandatory to avoid systemic comorbidities.

Recently, several studies have focused on the endothelial damage induced by intermittent hypoxia during sleep-disordered breathing. In particular, Abdolrahimzadeh et al., through the study of Optical Coherent Tomography (OCT), have shown a correlation between apnea/hypopnea episodes, oxidative stress and alterations in retinal blood flow, proposing these alterations as a non-invasive biomarker of endothelial damage [8].

Similarly, studies have been conducted to evaluate the possible consequences of OSA on the intestinal microbiota, showing how intermittent hypoxia—by altering normal intestinal permeability—creates imbalances in the microbiota in terms of microbial composition and abundance of species, the effects of which are still being studied [9,10,11].

Xu et al. have also shown how children with OSA show changes in the oral microbiota and present different metabolomic patterns compared to adults with OSA (in particular, metabolites associated with vitamin metabolism, the ornithine cycle, butanoate metabolism, and bilirubin metabolism), therefore suggesting that sleep-disordered breathing may have an effect on human metabolism [12].

Metabolomics is the comprehensive study of metabolites—the intermediate or end products of metabolic pathways—in a living organism, derived from biological fluids and tissues [13].

The metabolomic approach enables the early identification of individual metabolic responses to various endogenous and environmental pathophysiological stimuli, allowing a comprehensive understanding of the human metabolic processes and the development of therapeutic and management strategies that are specific and tailored to each patient [14].

Recently, metabolomics has emerged as a fundamental discipline in medical research, providing innovative insights across various medical fields, including pediatrics.

To date, few studies have analyzed the effects of sleep disorders in pediatric age from a metabolomic point of view.

The aim of this study is to analyze the metabolomic profile of children with sleep disorders to understand whether there are distinct and specific metabolic patterns related to the severity and type of sleep disorder (neurological or respiratory).

## 2. Methods

This is an observational, exploratory, hypothesis-generating pilot study conducted from January to February 2023 in the Pediatric Sleep Disease Centre at S. Andrea University Hospital, Sapienza University of Rome.

During that period, 12 patients, aged between 2 and 12 years, were enrolled after being referred to our department for sleep disorders, both neurological and breath-related sleep disturbances.

Patients with obesity (BMI > 97th percentile for ages 5–18; BMI > 99th percentile for ages 0–5), Down syndrome, Prader–Willi syndrome, other syndromic conditions, epilepsy, acute/chronic pulmonary or cardiac diseases, inflammatory bowel diseases, celiac disease, or known metabolic disorders were excluded from the study, as well as those who used probiotics, antibiotics, oral corticosteroids, or proton pump inhibitors within the previous month.

All enrolled patients received an anamnestic interview and a clinical evaluation to assess their overall health status and to collect biometric parameters (sex, age, height, weight, BMI). Then, they were hospitalized for two days to undergo a polysomnographic sleep study and urine collection for the metabolomic analysis. Patient Preparation: Morning urine samples were collected under fasting conditions, following at least 8 h of overnight rest and prior to physical activity, to avoid altering baseline metabolic levels. Participants were instructed to discard the first void and collect the subsequent urine sample. Dietary restrictions: In the 2–3 days preceding the test, patients are often required to avoid foods rich in polyphenols or compounds that mimic metabolites (e.g., bananas, kiwis, vanilla, chocolate and dried fruit). Parents were asked to properly wash their hands and external genitals before collecting the sample to avoid bacterial contamination.

The study was approved by the ethical board of the University of Rome Sapienza (Prot. 156 SA_2021 RIF. CE6266_2021) and was conducted in accordance with the tenets of Helsinki. Informed consent to participation in the study was obtained from the parents of all children.

### 2.1. Sleep Evaluation

First, all participants were given the validated Sleep Clinical Record (SCR) questionnaire to assess the presence of sleep-disordered breathing [15,16].

Then, during the first night of hospitalization, patients had an overnight oxygen saturation recording using a pulsoxymeter (Nonin 2500A, Nonin Medical, Plymouth, MN, USA). The recorded trace was next analyzed by technicians blinded to the SCR and polysomnography (PSG) results using PROXYnet 10.1 (MedicAir, Milan, Italy) software. Recorded traces lasting less than 6 h were considered invalid. Traces are scored according to the McGill score [17].

During the second night of hospitalization, children underwent an overnight polysomnography recorded with Grael Headbox V2 Compumedics (Melbourne, Australia) polygraphs and analyzed with Profusion PSG V4.5 software.

The variables recorded included an electroencephalogram with at least eight channels (frontal, central, temporal and occipital, referred to the contralateral mastoid), an electro-oculogram (electrodes placed 1 cm above the right outer cantus and 1 cm below the left outer cantus and referred to the mastoid electrode), and a submental electromyogram and an electrocardiogram (one derivation). Sleep was subdivided into 30 s epochs, and sleep stages were scored according to the standard criteria of the American Academy of Sleep Medicine (AASM). A digital time-synchronized video recording was performed. Chest and abdominal movements were measured by strain gauges. Oronasal airflow was recorded with a thermocouple and nasal pressure monitor when the child tolerated a nasal cannula. Arterial oxygen saturation was monitored with a pulse oximeter. Central, obstructive, and mixed apnea events and respiratory related arousals (RERAs) were counted according to the criteria established by the AASM.

Test started at the usual bedtime of the child and lasted until their spontaneous awakening the following morning. No medications were used to facilitate the patient’s falling asleep.

According to the standard criteria of the American Academy of Sleep Medicine [18], recorded traces were visually analyzed by an expert investigator blinded to the SCR score.

As for the respiratory aspect, the main parameters taken into consideration are the apnea hypopnea index (AHI), the oxygen desaturation index (ODI) and the wake after sleep onset (WASO). AHI is the number of obstructive apneas and hypopneas per hour of sleep. Obstructive sleep apnea (OSA) is diagnosed when AHI > 1 event/hour and its severity is classified as minimum (1–3 events/h), mild (3–5 events/h), moderate (5–10 events/h), and severe (>10 events/h). ODI is the average number of desaturations per hour of sleep, described as a decrease in the mean oxygen saturation of more than 3% for at least 10 s.

As for the neurological aspects of sleep disturbances, the EEG trace recorded during polysomnography was read by a pediatric neurologist to evaluate sleep architecture (Total Sleep Time, percentage of REM/NREM stages), to identify arousals and to differentiate between obstructive and central sleep apnea.

### 2.2. Urinalysis-Metabolomic Analysis

About 1 mL of each urine sample was transferred to a sterile test tube containing 8 µL of a 1% aqueous solution of NaN3 to inhibit bacteria growth and stored at −80 °C. The samples were subsequently prepared for Gas Chromatography Mass Spectrometry (GC-MS) analysis. A total of 150 μL of urine was transferred to a 2 mL Eppendorf tube with 200 μL of an aqueous urease solution (1 mg/mL) and subjected to ultrasound for 30 min. Then, 800 μL of methanol was added to denature the enzyme. After centrifugation, 750 μL of the supernatant was taken, transferred into glass test tubes, and evaporated to dryness in an Eppendorf vacuum centrifuge. The dried samples were then derivatized with 50 μL of a solution of methoxamine in pyridine (10 mg/mL; Sigma-Aldrich, St. Louis, MO, USA). After 1 h at 70 °C, 50 μL of N-Methyl-N-(trimethylsilyl) trifluoroacetamide (Sigma-Aldrich) was added, and the mixture was left to react at room temperature for 1 h. Then, samples were diluted with 600 µL of anhydrous hexane containing undecane as an internal standard. One µl of each sample was injected splitless into a 7890A gas chromatograph coupled with a 5975C mass spectrometer (Agilent Technologies, Santa Clara, CA, USA) equipped with a 30 m × 0.25 mm ID fused silica capillary column, with a 0.25 μM TG-5MS stationary phase (Thermo Fisher Scientific, Waltham, MA, USA). The injector temperature was 250 °C, the gas flow through the column was 1 mL/min, and the transfer line temperature was 280 °C. The column’s initial temperature was kept at 70 °C for 3 min, then increased to 250 °C at 12 °C/min and held for 4 min. Finally, the temperature was increased to 300 °C at 50 °C/min and kept for 1 min. Identification of metabolites was performed using the standard NIST 08 and GMD mass spectra libraries and, when available, by comparison with authentic standards [17]. Data processing was performed using MassHunter Software (Agilent Technologies, version 10.0). The total area of chromatograms (=100) was used to normalize the measurement of the metabolites from each urine sample.

### 2.3. Statistical Analysis

Statistical analyses were performed with JASP Version 0.19.3 (Apple Silicon, Cupertino, CA, USA).

Patients were divided into two groups: those with parasomnias (pavor) and those with sleep-disordered breathing. Clinical data (gender, age, and BMI) and sleep-related data were calculated and expressed using median and interquartile range (IQR) due to the small sample size and non-normal distribution of data. For this reason, statistical analyses between the two groups were conducted with the Mann–Whitney U test, considering *p* < 0.05 as statistically significant. Mean and standard deviation (SD) are also shown in Table 1 for descriptive purposes.

GraphPad Prism software (version 10; GraphPad Software, Inc., San Diego, CA, USA) was used to perform the univariate statistical analysis of the metabolomic data. Overall differences in metabolite concentrations among the three groups were assessed using the Kruskal–Wallis test. When the overall test was statistically significant, pairwise comparisons were performed using Dunn’s post hoc test with Bonferroni adjustment for the three pairwise comparisons conducted for each metabolite. Adjusted *p*-values < 0.05 were considered statistically significant. For metabolites showing a significant overall difference, the effect size was expressed as epsilon squared (ε^2^). Effect sizes for pairwise comparisons were expressed as Cliff’s delta (δ). Ninety-five percent confidence intervals for ε^2^ and Cliff’s δ were estimated using a stratified percentile bootstrap procedure with 10,000 resamples. Effect-size calculations and bootstrap confidence intervals were obtained using Python version 3.13.5. MetaboAnalyst 6.0 (https://www.metaboanalyst.ca, accessed on 27 May 2026) was used to perform enrichment and network analyses, respectively.

## 3. Results

### 3.1. Characteristics of Patients

One of the twelve enrolled patients was excluded due to a genetic diagnosis of epileptic encephalopathy.

The eleven patients eligible for the study were divided into two groups based on the type of sleep disorder, i.e., a group of patients suffering from sleep-disordered breathing (SDB) and a group of patients suffering from parasomnias (pavor nocturnus).

OSA patients were divided into mild (AHI between 1 and 5 ev/h) and moderate (AHI > 5 ev/h) patients while the third group was represented by patients with parasomnias.

Data are shown in Table 1.

### 3.2. Metabolomic Analysis

The urinary metabolomic profile of patients with OSA was analyzed using GC-MS.

The analysis allowed the identification of 70 metabolites, including amino acids, organic acids, sugars, and short-chain lipids. The concentration of the identified metabolites was subjected to a Kruskal–Wallis test in order to determine which metabolites were able to distinguish between the different degrees of OSA severity.

**Figure 1:**
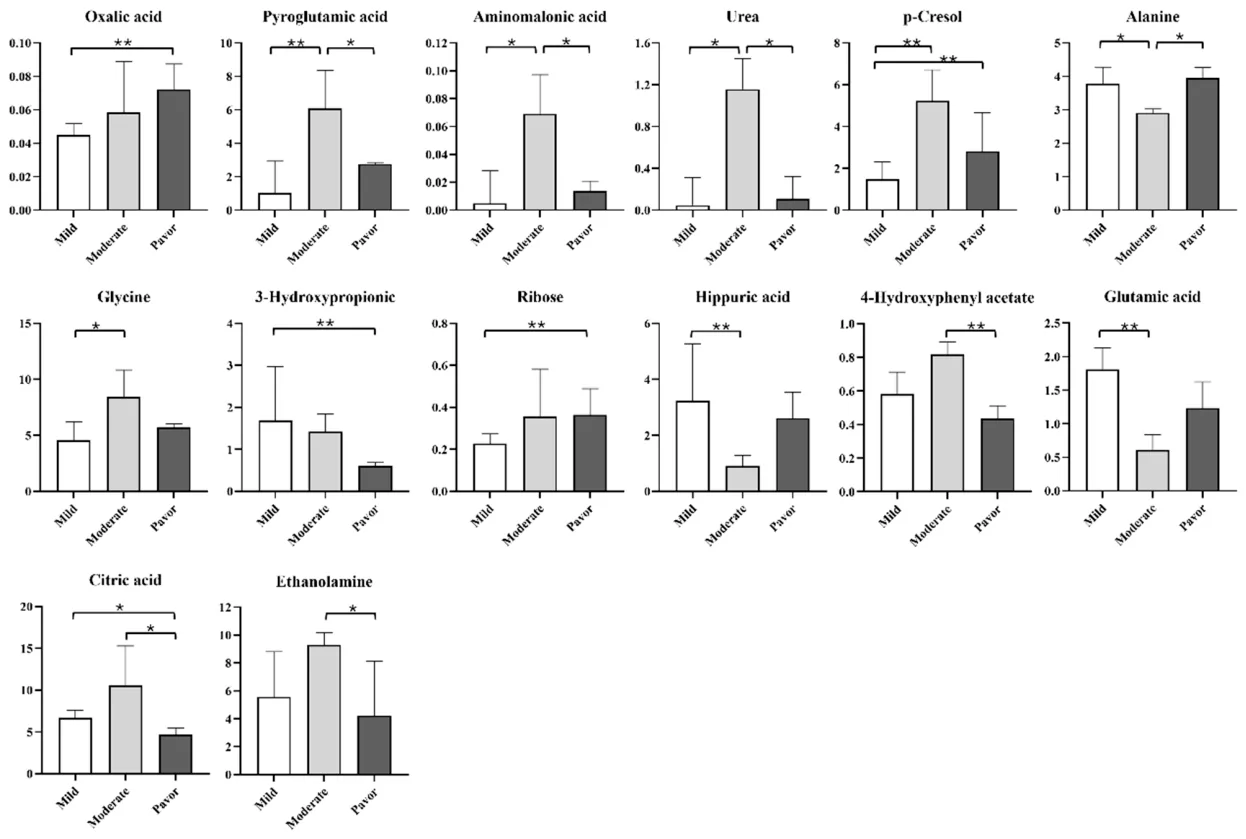
Bar plots illustrate the progressive change (relative concentration) in urine metabolite levels associated with varying degrees of OSAS severity. Statistical significance was determined Kruskal–Wallis test after Dunn’s adjustment. * *p*-value < 0.05; ** *p*-value <0.01.

**Figure 2:**
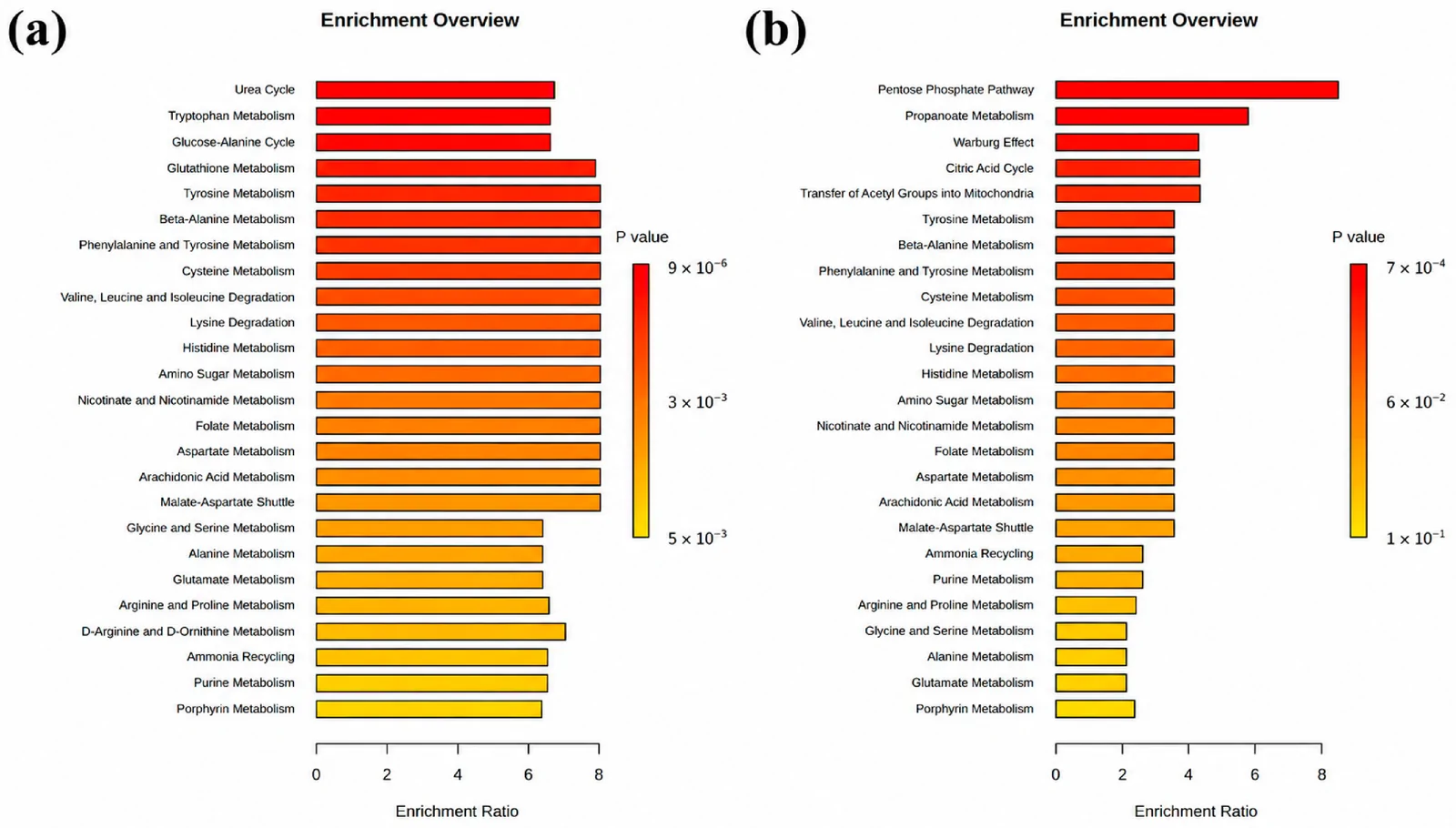
Enrichment analysis: list of the most significantly discriminant pathways between (**a**) mild and moderate patients and (**b**) mild and pavor patients.

The graph shown in Figure 1 includes only the statistically significant metabolites, with a *p*-value < 0.05. In detail, the oxalic acid and ribose increase in moderate OSA and pavor compared to mild patients, while 3-hydroxypropionic decrease. Furthermore, the pyroglutamic acid, aminomalonica acid, urea, p-cresol, glycine, 4-hydroxyphenyl acetate, citric acid and ethanolamine increase in moderate patients with respect to mild ones. Finally, hippuric acid and glutamic acid decrease in moderate patients compared to mild ones. The relative concentrations of discriminant metabolites and *p*-value values are reported in Table 2. The enrichment analysis revealed that urea cycle, tryptophan metabolism, glucose-alanine cycle, glutathione metabolism and beta-alanine metabolism were the most significantly discriminant pathways between mild and moderate patients (Figure 2a), while pentose phosphate pathway, propanoate metabolism, Warburg effect, citric acid cycle and transfer of acetyl group into mitochondria were the most significantly discriminant pathways between mild and pavor patients.

## 4. Discussion

Since this is one of the first studies concerning the application of metabolomics to investigate OSA and parasomnias, it was not easy to discuss each relevant metabolite found in our urine analysis.

Most of the metabolites found in our analysis are related to oxidative stress and systemic inflammation, known to be directly linked to sleep disturbances, as well as to the gut microbiota, which play a fundamental role in their production and function [19,20,21].

Oxalate derives from the metabolism of glycine that can be converted to glyoxylate, which in turn can be oxidized to oxalate.

Increased oxalate levels have been observed in individuals with OSA [22].

Indeed, augmented levels of this metabolite have been previously found in the urine or biofluids of patients suffering from depressive or anxiety disorders and sleep disorders, including OSA [22].

OSA causes intermittent hypoxia with subsequent activation of the sympathetic nervous system that could lead to chronic oxidative stress. Furthermore, these sleep alterations seem to alter various metabolisms, including that of ascorbate (vitamin C) and glyoxylate, leading to increased oxalate [23].

Moreover, oxalate can be produced or degraded by some intestinal bacteria (e.g., *Oxalobacter formigenes*). OSA and sleep disorders can alter the microbiota, thus also influencing the absorption and production of oxalate [24,25,26].

Ribose is a key metabolite in the pentose phosphate pathway (PPP). Its accumulation in urine may be due to the alterations of this pathway in patients with OSA caused especially by intermittent hypoxia, oxidative stress and sleep fragmentation. In particular, a proteomic study highlighted decreased PPP in red blood cells in patients with OSA [27].

In our sample, in contrast to our expectations based on the literature, we found a mild increase in these two metabolites in children with parasomnias compared to those with OSA. We hypothesize that this difference is due to the fact that these are studies conducted in adult patients with OSA. However, these data need to be verified through further studies with larger cohorts of patients.

3-hydroxypropionic acid is an intermediate organic acid that can be derived from the metabolism of amino acids such as threonine and valine, metabolism of propionate, mitochondrial dysfunction or hypoxia, or as a product of intestinal microbial fermentation [28].

Our urinary metabolomic analyses have shown a higher concentration of this metabolite in OSA patients when compared to the pavor ones.

There are no studies focused exclusively on this metabolite.

It is considered an indirect marker of oxidative stress, mitochondrial dysfunction, and alterations of the intestinal flora—all conditions associated with OSA [29].

Recent reviews confirm the importance of organic acids in the pathophysiology of OSA, although specific evidence for this acid is still emerging [30].

We observed increased levels of pyroglutamic acid (or 5-oxoproline) in patients with moderate OSA. This is an intermediate metabolite resulting from the degradation of glutathione and the increase in its levels in the presence of oxidative stress [31]. These findings support our data, in which pyroglutamic acid increased in patients with OSA, and, in particular, in those with a higher AHI. In fact, in oxidative stress induced by intermittent hypoxia, glutathione is one of the first cellular antioxidants that helps to neutralize this condition, and the increase in pyroglutamic acid derives from its degradation [32,33,34]. Excess pyroglutamic acid is eliminated in the urine, resulting in a higher urinary concentration.

Aminomalonic acid can be generated naturally via the activity of mammalian and bacterial enzymes on various precursors such as 2-aminomalonamide, diethylaminomalonate and ketomalonic acid.

Aminomalonic acid has also been found to be elevated in the urine of people with anxiety and major depressive disorders [35]. Since these psychological complications are more frequently observed in adults with OSA, it remains unclear why such metabolic alterations are present in our pediatric population, and further studies are necessary to clarify this finding. Furthermore, sleep fragmentation due to obstructive sleep apnea or parasomnia could be triggers for alterations in this metabolite.

Concerning urea, some metabolomics studies have observed an altered metabolic profile in patients with OSA, with changes in amino acid, urea, and nitrogenous compound levels and a tendency toward dysregulation of the urea cycle and protein metabolism linked to chronic intermittent hypoxia [36].

While, p-cresol is derived from the bacterial degradation of aromatic amino acids—particularly tyrosine and, to a lesser extent, phenylalanine—by the microbial flora [37].

It is also considered a uremic toxin and has been associated with renal damage and systemic toxic effects, especially in patients with chronic renal failure [38].

In our cohort, we found an increase in p-cresol levels in patients with moderate OSA; furthermore, in the literature, several studies demonstrate the presence of gut dysbiosis in OSA patients, with an increase in the species that produce this toxic compound. Indeed, even if there is no metabolomic study so far that has detected p-cresol in OSA patients, there is a microbiomic study in pediatric patients that highlighted the presence of dysbiosis [9,11].

Glycine is a nonessential amino acid with important roles in neurotransmission, modulation of inflammation, energy metabolism, and antioxidant function.

In some metabolomic studies on patients with OSA, increased glycine levels have been observed in biological samples (plasma, serum, or urine), likely in response to intermittent hypoxia, oxidative stress, systemic inflammation and alterations of gut microbiota [39].

Indeed, the intermittent hypoxia and chronic inflammation typical of OSA can cause functional impairment of the renal tubules, reducing the reabsorption capacity of amino acids such as glycine, resulting in increased urinary excretion.

Furthermore, intermittent hypoxia affects amino acid metabolism, leading to increased plasma glycine levels, which in turn are reflected in urine.

Finally, glycine has antioxidant and anti-inflammatory properties, so its urinary increase may also reflect compensatory cellular mechanisms aimed at counteracting oxidative stress [36].

However, there are no metabolomic or microbiomic studies on OSA yet that explicitly indicate 4-hydroxyphenylacetate (4-HPA) as a biomarker.

According to the Human Metabolome Database, 4-hydroxyphenylacetate is a metabolite of tyrosine, produced by both human enzymes and intestinal bacterial flora, and it can be present in urine, feces, blood, and saliva [40]. A possible explanation for detecting elevated 4-HPA levels in the urine of our OSA patients is that they may have an altered intestinal barrier (“leaky gut”), which could promote translocation of this metabolite and its urinary excretion [41,42,43]. However, given the limited data available and possible variability in urinary 4-HPA concentrations, this result remains preliminary [44].

In our study, urinary levels of citric acid were found to be increased in patients with obstructive sleep apnea. This result agrees with other evidence present in the literature, and several mechanisms may explain it [45].

First, intermittent hypoxia in OSA can lead to mitochondrial hyperactivity and oxidative stress, which may result in the increased production and excretion of citrate [46,47].

Second, renal citrate excretion is modulated by systemic acid–base balance [48].

In some OSA patients—especially early-stage or metabolically compensated cases—renal handling of citrate may shift toward enhanced excretion, particularly in the absence of systemic acidosis.

Lastly, a subclinical proximal tubular dysfunction may contribute to reduced citrate reabsorption, a phenomenon described in OSA-related kidney impairment in adult patients [49].

Overall, elevated urinary citrate might serve as an early marker of metabolic or renal alterations in OSA.

Ethanolamine, a key metabolite in phospholipid turnover, was found to be increased in OSA, caused by sleep fragmentation—confirming our result [44]. This may reflect altered membrane remodeling, mitochondrial stress, or low-grade systemic inflammation. Though not a primary biomarker of OSA, ethanolamine could represent a secondary indicator of cellular stress in the context of repeated episodes of intermittent hypoxia.

In our study, urinary levels of hippuric acid were significantly lower in patients with moderate OSA compared to those with mild disease. Hippuric acid is a hepatic conjugation product of benzoic acid and glycine and reflects both the microbial metabolism of dietary aromatic compounds and liver-phase II detoxification capacity [50,51]. Its reduction may indicate impaired hepatic mitochondrial function due to sustained intermittent hypoxia, alterations in gut microbiota-derived benzoate availability, or reduced systemic glycine pools. Our findings, in line with others in the literature, may suggest that progressive OSA and its related gut microbiota alterations could be associated with early metabolic dysregulation involving aromatic compound clearance in these patients [52].

In our study, urinary glutamic acid levels were significantly reduced in moderate OSA patients, while patients with pavor and with mild obstructive sleep apnea have higher levels of this metabolite. A dysregulation of the GABAergic/glutamatergic system could lead to alteration in sleep control and neurodevelopmental diseases and altered cognitive functions [51].

This finding may reflect altered amino acid metabolism under chronic intermittent hypoxia, increased glutamate utilization for glutathione synthesis during oxidative stress, or a neuroprotective shift limiting extracellular excitotoxicity [53]. The associations observed between metabolite profiles and disease phenotype, as well as the corresponding pathway enrichment, should be interpreted in the context of the limited sample size and are intended primarily to generate hypotheses for further investigation.

## 5. Strength and Limitations

Our pilot study is the first to investigate metabolomics in children with obstructive sleep apnea and pavor. However, several limitations must be acknowledged.

The small overall sample size and, particularly, the limited number of participants within the moderate OSA and pavor subgroups, may have resulted in imprecise effect-size estimates and wide confidence intervals. Therefore, although effect sizes and their 95% confidence intervals are now reported, they should be interpreted cautiously and considered exploratory rather than stable or generalizable estimates. Furthermore, in our study, gut microbiota was not analyzed, even though it is known to play a crucial role in human metabolic processes and would provide a better contextualization of the data obtained.

## 6. Conclusions

In conclusion, despite the limited sample size, our data reveal interesting preliminary associations between metabolomics and sleep disorders. These insights deserve further investigation in larger cohorts of patients and integrating different approaches (e.g., metabolomics, oxidative stress analysis, microbiota profiling…) to enhance our understanding of the field and guide clinicians toward precise and personalized patient management.

## Figures and Tables

**Table 1:** Characteristics of the participants.

	Mild OSA (n = 5)	Moderate OSA (n = 3)	Parasomnias (n = 3)		*p* Value	
				Mild vs. Moderate	Mild vs. Parasomnias	Moderate vs. Parasomnias
Male sex n (%)	4 (80%)	2 (66.6%)	1 (33%)	0.673	0.19	0.414
Mean age (yr)	6.64 ± 4.3	5.0 ± 2.1	9.4 ± 3.1	0.764	0.297	0.127
BMI (kg/m^2^)	16.3 (15.4–30.7)	15.8 (15.6–18.7)	19.3 (14.8–21.5)	0.770	0.881	0.513
SCR (score)	7 (6.5–7.0)	8 (7.0–8.5)	4 (3.0–4.2)	0.055	**0.021**	**0.050**
AHI (ev/h)	3.9 (1.6–4.4)	5.3 (5.1–7.3)	0.5 (0.3–0.6)	**0.025**	**0.025**	**0.050**
Mean SaO_2_ (%)	97.8 (97.5–98.1)	97.3 (97.2–98.2)	99 (98.9–99)	0.370	**0.022**	**0.037**
Minimal SaO_2_ (%)	95.2 (93.6–95.8)	95.1 (84.6–95.8)	96.5 (96.2–96.7)	0.881	**0.050**	**0.050**

**Table 2:** Relative concentrations of discriminant metabolites.

Metabolite	Relative Concentration, Median (IQR)			Overall Comparison		Dunn Pairwise Comparisons: Adjusted *p*; Cliff’s δ (95% CI)		
	Mild OSA	Moderate OSA	Pavor	Kruskal–Wallis *p*	ε^2^ (95% CI)	Mild vs. Moderate	Mild vs. Pavor	Moderate vs. Pavor
Oxalic acid	0.045 (0.039–0.049)	0.058 (0.048–0.074)	0.072 (0.070–0.082)	**0.011**	0.41 (0.18 to 0.75)	*p* = 0.472; δ = −0.50 (−1.00 to 0.20)	***p*** ** = 0.009;** **δ = −0.90 (−1.00 to −0.60)**	*p* = 0.855; δ = −0.42 (−1.00 to 0.50)
Pyroglutamic acid	1.008 (0.459–2.596)	6.095 (4.524–7.867)	2.754 (1.296–2.836)	**0.009**	0.43 (0.42 to 0.66)	***p*** ** = 0.008;** **δ = −1.00 (−1.00 to −1.00)**	*p* = 1.000; δ = −0.17 (−0.73 to 0.43)	***p*** ** = 0.049;** **δ = 1.00 (1.00 to 1.00)**
Aminomalonic acid	0.005 (0.001–0.023)	0.069 (0.045–0.092)	0.014 (0.003–0.015)	**0.018**	0.36 (0.21 to 0.63)	***p*** ** = 0.020;** **δ = −0.95 (−1.00 to −0.80)**	*p* = 1.000; δ = −0.03 (−0.70 to 0.67)	***p*** ** = 0.049;** **δ = 0.92 (0.58 to 1.00)**
Urea	0.047 (0.028–0.242)	1.158 (1.124–1.258)	0.106 (0.044–0.278)	**0.030**	0.29 (0.11 to 0.58)	***p*** ** = 0.039;** **δ = −0.80 (−1.00 to −0.40)**	*p* = 1.000; δ = −0.03 (−0.67 to 0.57)	*p* = 0.062; δ = 1.00 (1.00 to 1.00)
p-cresol	1.474 (0.407–2.168)	5.227 (3.475–6.571)	2.811 (2.554–4.194)	**0.004**	0.54 (0.26 to 0.83)	***p*** ** = 0.017;** **δ = −0.85 (−1.00 to −0.40)**	***p*** ** = 0.021;** **δ = −0.90 (−1.00 to −0.60)**	*p* = 1.000; δ = 0.33 (−0.50 to 1.00)
Alanine	3.782 (3.540–3.948)	2.906 (2.827–2.988)	3.958 (3.740–4.196)	**0.010**	0.43 (0.42 to 0.63)	***p*** ** = 0.018;** **δ = 1.00 (1.00 to 1.00)**	*p* = 1.000; δ = −0.13 (−0.70 to 0.47)	***p*** ** = 0.016;** **δ = −1.00 (−1.00 to −1.00)**
Glycine	4.546 (4.011–5.925)	8.433 (6.083–10.788)	5.691 (5.610–5.934)	**0.031**	0.29 (0.03 to 0.75)	***p*** ** = 0.026;** **δ = −0.75 (−1.00 to −0.25)**	*p* = 1.000; δ = −0.40 (−0.87 to 0.20)	*p* = 0.292; δ = 0.92 (0.50 to 1.00)
3-Hydroxy propionic	1.681 (0.966–2.843)	1.420 (1.006–1.836)	0.612 (0.403–0.670)	**0.002**	0.60 (0.59 to 0.74)	*p* = 1.000; δ = 0.20 (−0.40 to 0.80)	***p*** ** = 0.002;** **δ = 1.00 (1.00 to 1.00)**	*p* = 0.055; δ = 1.00 (1.00 to 1.00)
Ribose	0.227 (0.164–0.265)	0.355 (0.130–0.578)	0.364 (0.347–0.449)	**0.027**	0.31 (0.29 to 0.83)	*p* = 0.871; δ = −0.10 (−1.00 to 0.80)	***p*** ** = 0.022;** **δ = −1.00 (−1.00 to −1.00)**	*p* = 0.716; δ = 0.00 (−1.00 to 1.00)
Hippuric acid	3.236 (3.114–4.805)	0.900 (0.821–1.073)	2.614 (1.848–3.371)	**0.021**	0.33 (0.13 to 0.69)	***p*** ** = 0.018;** **δ = 0.90 (0.55 to 1.00)**	*p* = 0.594; δ = 0.43 (−0.17 to 0.90)	*p* = 0.414; δ = −0.67 (−1.00 to 0.00)
4-Hydroxyphenylacetate	0.582 (0.521–0.661)	0.819 (0.759–0.881)	0.437 (0.418–0.475)	**0.011**	0.42 (0.24 to 0.73)	*p* = 0.128; δ = −0.80 (−1.00 to −0.40)	*p* = 0.449; δ = 0.50 (−0.07 to 1.00)	***p*** ** = 0.008;** **δ = 1.00 (1.00 to 1.00)**
Glutamic acid	1.809 (1.666–1.851)	0.612 (0.403–0.825)	1.233 (1.214–1.394)	**0.002**	0.62 (0.43 to 0.84)	***p*** ** = 0.002;** **δ = 1.00 (1.00 to 1.00)**	*p* = 0.200; δ = 0.70 (0.10 to 1.00)	*p* = 0.266; δ = −1.00 (−1.00 to −1.00)
Citric acid	6.702 (6.097–7.441)	10.530 (5.796–15.136)	4.707 (3.924–5.332)	**0.017**	0.36 (0.10 to 0.73)	*p* = 1.000; δ = −0.25 (−1.00 to 0.60)	***p*** ** = 0.040;** **δ = 0.80 (0.40 to 1.00)**	***p*** ** = 0.041;** **δ = 0.83 (0.33 to 1.00)**
Ethanolamine	5.569 (5.154–8.266)	9.307 (8.708–9.871)	4.234 (3.847–7.197)	**0.033**	0.28 (0.07 to 0.64)	*p* = 0.184; δ = −0.70 (−1.00 to −0.20)	*p* = 0.811; δ = 0.37 (−0.23 to 0.90)	***p*** ** = 0.028;** **δ = 0.92 (0.50 to 1.00)**

Data are expressed as median (interquartile range). Kruskal–Wallis *p*-values refer to the overall comparison among the three groups. Dunn *p*-values were adjusted across the three pairwise comparisons within each metabolite using the Bonferroni method. ε^2^ denotes epsilon squared. Cliff’s δ is oriented according to the order shown in each column; positive values indicate higher concentrations in the first-listed group. Ninety-five percent confidence intervals were estimated using 10,000 stratified bootstrap resamples.

## Data Availability

The original contributions presented in this study are included in the article. Further inquiries can be directed to the corresponding author.

## Abbreviations

OSA	Obstructive Sleep Apnea
ROS	Reactive Oxygen Species
OCT	Optical Coherent Tomography
SCR	Sleep Clinical Record
PSG	Polysomnography
AHI	Apnea Hypopnea Index
ODI	Oxygen Desaturation Index
WASO	Wake After Sleep Onset
SD	Standard Deviation
IQR	Interquartile Range
GC-MS	Gas Chromatography Mass Spectrometry
PPP	Pentose Phosphate Pathway
4-HPA	4-hydroxyphenylacetate

## References

[1] Maski K., Owens J. (2018). Pediatric Sleep Disorders. Continuum.

[2] Piotto M., Gambadauro A., Rocchi A., Lelii M., Madini B., Cerrato L., Chironi F., Belhaj Y., Patria M.F. (2023). Pediatric Sleep Respiratory Disorders: A Narrative Review of Epidemiology and Risk Factors. Children.

[3] Singh S., Kaur H., Singh S., Khawaja I. (2018). Parasomnias: A Comprehensive Review. Cureus.

[4] Bruni O., DelRosso L.M., Melegari M.G., Ferri R. (2024). The Parasomnias. Psychiatr. Clin. N. Am..

[5] Garde A.J., Gibson N.A., Samuels M.P., Evans H.J. (2022). Recent advances in paediatric sleep disordered breathing. Breathe.

[6] Yu P.K., Radcliffe J., Taylor H.G., Amin R.S., Baldassari C.M., Boswick T., Chervin R.D., Elden L.M., Furth S.L., Garetz S.L. (2022). Neurobehavioral morbidity of pediatric mild sleep-disordered breathing and obstructive sleep apnea. Sleep.

[7] Lei L., Zhang X., Wang B., Lei F., Dai L., Sun X., Zhao Y., Zhu P., Zou J. (2024). Effects of sleep-disordered breathing on serum lipid levels in children:a case control study. BMC Pediatr..

[8] Abdolrahimzadeh S., Evangelisti M., Gattazzo I., Arpinelli M., Di Nardo G., Federico D.S., Simmaco M., Salerno G., Parisi P., Scuderi G. (2023). Oxidative stress and optical coherence tomography angiography evaluation of choriocapillaris and retinal vessel density in children with obstructive sleep apnea. Sleep Breath..

[9] da Silva L.M.A.V., Assunção W.G., Bento V.A.A., Sachi V.P., Colombo F.E., Ique M.M.A., Faria B.M.A., Bertoz A.P.d.M. (2024). Assessment of the gut microbiota of children with obstructive sleep apnea syndrome: A systematic review. Sleep Med..

[10] Moreno-Indias I., Torres M., Montserrat J.M., Sanchez-Alcoholado L., Cardona F., Tinahones F.J., Gozal D., Poroyko V.A., Navajas D., Queipo-Ortuño M.I. (2014). Intermittent hypoxia alters gut microbiota diversity in a mouse model of sleep apnoea. Eur. Respir. J..

[11] Valentini F., Evangelisti M., Arpinelli M., Di Nardo G., Borro M., Simmaco M., Villa M.P. (2020). Gut microbiota composition in children with obstructive sleep apnoea syndrome: A pilot study. Sleep Med..

[12] Xu H., Li X., Zheng X., Xia Y., Fu Y., Li X., Qian Y., Zou J., Zhao A., Guan J. (2018). Pediatric obstructive sleep apnea is associated with changes in the oral microbiome and urinary metabolomics profile: A pilot study. J. Clin. Sleep Med..

[13] Noto A., Fanos V., Dessì A. (2016). Metabolomics in Newborns. Advances in Clinical Chemistry.

[14] Bardanzellu F., Fanos V. (2020). How could metabolomics change pediatric health?. Ital. J. Pediatr..

[15] Villa M.P., Paolino M.C., Castaldo R., Vanacore N., Rizzoli A., Miano S., Del Pozzo M., Montesano M. (2012). Sleep clinical record: An aid to rapid and accurate diagnosis of paediatric sleep disordered breathing. Eur. Respir. J..

[16] Villa M.P., Sujanska A., Vitelli O., Evangelisti M., Rabasco J., Pietropaoli N., Banovcin P., Kheirandish-Gozal L., Gozal D. (2015). Use of the sleep clinical record in the follow-up of children with obstructive sleep apnea (OSA) after treatment. Sleep Breath..

[17] Trucco F., Rosenthal M., Bush A., Tan H.-L. (2020). The McGill score as a screening test for obstructive sleep disordered breathing in children with co-morbidities. Sleep Med..

[18] Marcus C.L., Brooks L.J., Draper K.A., Gozal D., Halbower A.C., Jones J., Schechter M.S., Ward S.D., Sheldon S.H., Shiffman R.N. (2012). Diagnosis and management of childhood obstructive sleep apnea syndrome. Pediatrics.

[19] Spada M., Piras C., Diana G., Leoni V.P., Frau D.V., Serreli G., Simbula G., Loi R., Noto A., Murgia F. (2023). Glutamine Starvation Affects Cell Cycle, Oxidative Homeostasis and Metabolism in Colorectal Cancer Cells. Antioxidants.

[20] Vernocchi P., Del Chierico F., Putignani L. (2016). Gut microbiota profiling: Metabolomics based approach to unravel compounds affecting human health. Front. Microbiol..

[21] Conlon M.A., Bird A.R. (2014). The impact of diet and lifestyle on gut microbiota and human health. Nutrients.

[22] Du D., Luo J., Cai W., Qin J., Yang Y., Hu X., Li X., Luo F., Shen Y. (2024). Self-Reported Symptoms of Obstructive Sleep Apnea are Associated with Increased Risk of Kidney Stones: A Cross-Sectional Study from NHANES 2015–2020. Nat. Sci. Sleep.

[23] Tallman J.E., Stone B.V., Sui W., Miller N.L., Hsi R.S. (2023). Association between obstructive sleep apnea and 24-h urine chemistry risk factors for urinary stone disease. Urolithiasis.

[24] Xue J., Allaband C., Zuffa S., Poulsen O., Meadows J., Zhou D., Dorrestein P.C., Knight R., Haddad G.G. (2025). Gut microbiota and derived metabolites mediate obstructive sleep apnea induced atherosclerosis. Gut Microbes.

[25] Smith R.P., Easson C., Lyle S.M., Kapoor R., Donnelly C.P., Davidson E.J., Parikh E., Lopez J.V., Tartar J.L. (2019). Gut microbiome diversity is associated with sleep physiology in humans. PLoS ONE.

[26] Wang F., Zou J., Xu H., Huang W., Zhang X., Wei Z., Li X., Liu Y., Zou J., Liu F. (2022). Effects of Chronic Intermittent Hypoxia and Chronic Sleep Fragmentation on Gut Microbiome, Serum Metabolome, Liver and Adipose Tissue Morphology. Front. Endocrinol..

[27] Valentim-Coelho C., Saraiva J., Osório H., Antunes M., Vaz F., Neves S., Pinto P., Bárbara C., Penque D. (2025). Red blood cell proteomic profiling in mild and severe obstructive sleep apnea patients before and after positive airway pressure treatment. Biochim. Biophys. Acta (BBA) —Mol. Basis Dis..

[28] Vidra A., Németh Á. (2017). Bio-based 3-hydroxypropionic Acid: A Review. Period. Polytech. Chem. Eng..

[29] Kildegaard K.R., Hallström B.M., Blicher T.H., Sonnenschein N., Jensen N.B., Sherstyk S., Harrison S.J., Maury J., Herrgård M.J., Juncker A.S. (2014). Evolution reveals a glutathione-dependent mechanism of 3-hydroxypropionic acid tolerance. Metab. Eng..

[30] Mohit Tomar M.S., Araniti F., Pateriya A., Singh Kushwaha R.A., Singh B.P., Jurel S.K., Singh R.D., Shrivastava A., Chand P. (2022). Identification of metabolic fingerprints in severe obstructive sleep apnea using gas chromatography–Mass spectrometry. Front. Mol. Biosci..

[31] Turathum B., Gao E.-M., Yang F., Liu Y.-B., Yang Z.-Y., Liu C.-C., Xue Y.-J., Wu M.-H., Wang L., Grataitong K. (2022). Role of pyroglutamic acid in cumulus cells of women with polycystic ovary syndrome. J. Assist. Reprod. Genet..

[32] Mohit Tomar M.S., Sharma D., Nandan S., Pateriya A., Shrivastava A., Chand P. (2022). Emerging role of metabolomics for biomarker discovery in obstructive sleep apnea. Sleep Breath..

[33] Kumar A., Bachhawat A.K. (2012). Pyroglutamic Acid: Throwing Light on a Lightly Studied Metabolite. Curr. Sci..

[34] Gueta I., Ovadia Y.P., Markovits N., Schacham Y.N., Epsztein A., Loebstein R. (2020). Is Pyroglutamic Acid a Prognostic Factor Among Patients with Suspected Infection? A Prospective Cohort Study. Sci. Rep..

[35] Chen J.-J., Bai S.-J., Li W.-W., Zhou C.-J., Zheng P., Fang L., Wang H.-Y., Liu Y.-Y., Xie P. (2018). Urinary biomarker panel for diagnosing patients with depression and anxiety disorders. Transl. Psychiatry.

[36] Xu H., Zheng X., Qian Y., Guan J., Yi H., Zou J., Wang Y., Meng L., Zhao A., Yin S. (2016). Metabolomics Profiling for Obstructive Sleep Apnea and Simple Snorers. Sci. Rep..

[37] Smith E., Macfarlane G. (1997). Formation of Phenolic and Indolic Compounds by Anaerobic Bacteria in the Human Large Intestine. Microb. Ecol..

[38] Ramezani A., Raj D.S. (2014). The gut microbiome, kidney disease, and targeted interventions. J. Am. Soc. Nephrol..

[39] Kiens O., Taalberg E., Ivanova V., Veeväli K., Laurits T., Tamm R., Ottas A., Kilk K., Soomets U., Altraja A. (2021). The effect of obstructive sleep apnea on peripheral blood amino acid and biogenic amine metabolome at multiple time points overnight. Sci. Rep..

[40] Semba R.D., Trehan I., Li X., Moaddel R., Ordiz M.I., Maleta K.M., Kraemer K., Shardell M., Ferrucci L., Manary M. (2017). Environmental Enteric Dysfunction is Associated with Carnitine Deficiency and Altered Fatty Acid Oxidation. EBioMedicine.

[41] Li Q., Xu T., Zhong H., Gao W., Shao C., Feng L., Li T. (2020). Impaired intestinal barrier in patients with obstructive sleep apnea. Sleep Breath..

[42] Gawlik-Kotelnicka O., Margulska A., Gabryelska A., Sochal M., Białasiewicz P., Strzelecki D. (2022). “Leaky Gut” as a Keystone of the Connection between Depression and Obstructive Sleep Apnea Syndrome? A Rationale and Study Design. Metabolites.

[43] Wang X., Li Y., Wang X., Wang R., Hao Y., Ren F., Wang P., Fang B. (2024). Faecalibacterium prausnitzii Supplementation Prevents Intestinal Barrier Injury and Gut Microflora Dysbiosis Induced by Sleep Deprivation. Nutrients.

[44] Humer E., Pieh C., Brandmayr G. (2020). Metabolomics in sleep, insomnia and sleep apnea. Int. J. Mol. Sci..

[45] Giskeødegård G.F., Davies S.K., Revell V.L., Keun H., Skene D.J. (2015). Diurnal rhythms in the human urine metabolome during sleep and total sleep deprivation. Sci. Rep..

[46] Lavie L. (2015). Oxidative stress in obstructive sleep apnea and intermittent hypoxia—Revisited—The bad ugly and good: Implications to the heart and brain. Sleep Med. Rev..

[47] Li C., Shi S. (2024). Gut microbiota and metabolic profiles in chronic intermittent hypoxia-induced rats: Disease-associated dysbiosis and metabolic disturbances. Front. Endocrinol..

[48] Hamm L., Hering-Smith K.S. (2002). Pathophysiology of Hypocitraturic Nephrolithiasis. Endocrinol. Metab. Clin. N. Am..

[49] Hou Y., Li Y., Xiao Z., Wang Z. (2024). Causal effects of obstructive sleep apnea on chronic kidney disease and renal function: A bidirectional Mendelian randomization study. Front. Neurol..

[50] Nicholls A.W., Mortishire-Smith R.J., Nicholson J.K. (2003). NMR Spectroscopic-Based Metabonomic Studies of Urinary Metabolite Variation in Acclimatizing Germ-Free Rats. Chem. Res. Toxicol..

[51] Yavuz A., Tetta C., Ersoy F.F., D’intini V., Ratanarat R., De Cal M., Bonello M., Bordoni V., Salvatori G., Andrikos E. (2005). Uremic toxins: A new focus on an old subject. Semin. Dial..

[52] Koundal S., Gandhi S., Kaur T., Mazumder A., Khushu S. (2015). “Omics” of high altitude biology: A urinary metabolomics biomarker study of rats under hypobaric hypoxia. OMICS J. Integr. Biol..

[53] Kaczmarski P., Sochal M., Strzelecki D., Białasiewicz P., Gabryelska A. (2023). Influence of glutamatergic and GABAergic neurotransmission on obstructive sleep apnea. Front. Neurosci..

